# Sexting Among Adolescents: The Emotional Impact and Influence of the Need for Popularity

**DOI:** 10.3389/fpsyg.2019.01828

**Published:** 2019-08-21

**Authors:** Rosario Del Rey, Mónica Ojeda, José A. Casas, Joaquín A. Mora-Merchán, Paz Elipe

**Affiliations:** ^1^Department of Educational and Developmental Psychology, Universidad de Sevilla, Seville, Spain; ^2^Department of Psychology, Universidad de Córdoba, Cordoba, Spain; ^3^Department of Psychology, Universidad de Jaén, Jaen, Spain

**Keywords:** sexting, emotional impact, popularity, adolescents, risk factors

## Abstract

Sexting refers to the exchange of sexual content material *via* technological devices. The definitions of this phenomenon vary greatly, mainly, depending on the types of sexting: primary and secondary. Besides the above, there is no common perspective on whether sexting is a risk behavior that entails some type of impact by itself or not and, in such a case, whether this impact varies according to gender. In addition, the need to be popular has shown to be a factor that could increase the probability of being involved in sexting. The present study analyzes the potential emotional impact of sexting as well as the effect of the need for popularity on this phenomenon and if it varies according to gender. The sample comprised 2,356 high school students (46.8% female, 53.2% male; age range 11–18 years old, *M* = 13.72; SD = 1.31) belonging to 12 compulsory secondary education (ESO) schools from the south of Spain. To assess sexting implication, four questions were presented to participants (sending, receiving, forwarding, and receiving sexts *via* intermediary). Scales, self-report, about emotional impact (depressed, annoyed, and active) and need for popularity were also applied. The results obtained show that, although sexting has a clear emotional impact on adolescents, it does not appear to generate a negative impact among those involved, at least in the short term. Concretely, this phenomenon seems to trigger emotions related to activation in boys and girls (I feel lively, energetic, satisfied, ready, determined, active). Additionally, with respect to the need for popularity, its relevance, specially, in relation to active emotional impact has been confirmed by the analyses. Statistical models found for boys and girls were similar. In addition, some differences in emotional impact by gender were found, girls feeling more depressed and annoyed in secondary sexting, and boys more active regarding both types of sexting.

## Introduction

The digital world has opened up a host of opportunities in adolescent social life. The use of electronic media for sharing and exchanging content of a sexual nature has become another form of intimate sexual communication attuned to today’s technology-driven society (Döring, 2014). In general terms, sexting refers to the exchange of sexual material *via* a technological device (Van Ouytsel et al., 2015). However, sexting definitions vary much depending on the behavior in question, the type of material, and whether sexting is restricted to sexual content or also encompasses erotic content (Barrense-Dias et al., 2017). The conceptual delimitations range from more restrictive ones which exclusively identify sexting as sending one’s own sexually explicit images (Wolak et al., 2012; Ybarra and Mitchell, 2014; Choi et al., 2016; Marume et al., 2018) to more comprehensive ones which include other behaviors that cover the dissemination of sexual content to third parties, such as sending, receiving, and forwarding sexually suggestive and explicit photos, videos, and text messages (Mitchell et al., 2012; Villacampa, 2017). The different between these two kind of conceptual delimitations support the categorization of primary and secondary sexting (Calvert, 2009; Schmitz and Siry, 2011). In the first case, minors send sexts between two people and do not share any further. In secondary sexting, someone shares sexts and they are forwarded beyond the intended recipient. It is relevant to mention that primary sexting tends to be consensual (with some exceptions like sextortion), but secondary sexting is likely to be non-consensual having a greater impact (Lievens, 2014), when freedom of choice is sometimes not an option (Walker and Sleath, 2017).

Involvement rates are highly varied, largely because of the wide range of attributable meanings (Barrense-Dias et al., 2017). When sexting is defined as the sending of sexual content, prevalence ranges from 4.6 (Rice et al., 2012) to 31% (Woodward et al., 2017). Receiving rates go anywhere from 7.1 (Mitchell et al., 2012) to 49% (Lippman and Campbell, 2014; Woodward et al., 2017), whereas prevalence rates for forwarding sexual content range from 2.3 (Lippman and Campbell, 2014) to 25% (Strassberg et al., 2017). These variations are partly linked to the increasing frequency of sexting in recent years (Clancy et al., 2019). Research has also pointed out how sexting is increasing with age (Madigan et al., 2018), especially among adolescents (Klettke et al., 2014). However, the onset of sexting could be starting earlier as age of access to smartphones is decreasing (Influence Central, 2016). This circumstance makes it necessary to develop more studies to analyze sexting behaviors in young adolescents.

To date, studies have failed to show a clear pattern of results concerning possible gender differences in relation to sexting prevalence. Some studies report that girls are more likely to share sexual images than boys (Reyns et al., 2013; Ybarra and Mitchell, 2014); other studies find boys participating more in this activity (West et al., 2014; Gámez-Guadix et al., 2017); and some studies observe no gender differences in sending and receiving sexual photos and messages (Lenhart, 2009; Rice et al., 2012; Campbell and Park, 2014; Vanden Abeele et al., 2014). These differences, in one direction or another, could be due to the type of sexting behavior being analyzed. As such, researchers have found that boys forward and request sexual photos and messages to a greater degree than girls, and that girls acknowledge that content of this type is more frequently asked of them (Norman, 2017; Symons et al., 2018). Ringrose et al. (2013) have pointed out that gender differences in sexting behavior can also be linked to differences in motivations for sexting. Thus, whereas sexting seems to increase status in boys, girls’ participation in sexting causes feeling of shame about themselves and their sexual reputation establishing what has been identified as a sexual double standard (Ringrose et al., 2013).

Apart from the sexting involvement rates, this phenomenon has attracted increased public and scientific attention in recent years because of its potential consequences (Gewirtz-Meydan et al., 2018). However, not everyone in the scientific community considers sexting a risk behavior (de Souza and Alves Banaco, 2018). Some authors defend adolescents’ freedom of sexual expression *via* the Internet, arguing that the risks associated with this behavior do not lie in the transfer of files itself, but with the potentiality of its quick and widespread dissemination, thus widening the target audience (Livingstone and Görzig, 2014). However, other studies have found that sexting can affect the physical and psychological health of those involved as well as trigger symptoms of depression and even suicidal ideation (Strasburger et al., 2012; Jasso Medrano et al., 2018). Besides, sexting has also demonstrated to be associated to other risk behaviors (e.g., cyberpornography; Morelli et al., 2017). Therefore, we agree with those authors who consider it is necessary to act upon any potentially risky online behaviors, and, in this case, the very behavior of sexting can have an impact in itself (Van Ouytsel et al., 2014a). Therefore, sexting may bring an emotional impact and negative consequences for those involved (Klettke et al., 2014; Van Ouytsel et al., 2015; Choi et al., 2016). The reasons given for this phenomenon’s potential impact include the transgression of sexual boundaries and the non-consensual distribution of sexual content to third parties (Dekker and Thula, 2017). Impact is also linked to different motivations (sexual, instrumental, and body image reinforcement) of sexting behavior, being instrumental reasons which cause higher negative impact (Bianchi et al., 2018). In addition, previous studies have shown how online victimization is associated to negative emotional impact (Ortega et al., 2012; Slonje et al., 2017). It would be necessary to confirm if the emotional impact of sexting is following the same negative pattern as other forms of online aggression/victimization (Giménez Gualdo et al., 2015). Concerning gender, there are also some studies stating a differential emotional impact depending on victims’ gender, usually pointing out the higher negative impact in girls (Bastomski and Smith, 2017; Betts et al., 2019).

Although most studies analyze sending and/or receiving, primary sexting, it seems that the action most likely to pose greater harm and, therefore, play a more important role in understanding the consequences behind this phenomenon is the action of forwarding (Livingstone and Görzig, 2014; Dekker and Thula, 2017; Strassberg et al., 2017). The forwarding of sexual content refers to sending someone else’s material to another person (Strassberg et al., 2017), secondary sexting, usually done without consent, which increases the risks of damaging the reputation of the victim (Van Ouytsel et al., 2014b), and increases the risk of being involved in dating violence episodes (Morelli et al., 2016).

The consequences of sexting seem to affect boys and girls differently. It is usually more harmful for girls, as they tend to be at the receiving end of more insults and humiliation, thus damaging their reputation (Wood et al., 2015). In turn, boys can experience positive consequences; for example, increased acceptance inside peer group (Speno, 2016; Burén and Lunde, 2018). This reality exposes the sexual double standard governing sexting, as it is girls who are more likely to have their reputation tarnished and who mostly face the consequences of this phenomenon as well as a greater negative impact (Wood et al., 2015). Thus, there seem to be different patterns to explain the roles that boys and girls take on in the negotiation process and the consequences by gender (Wood et al., 2015; Symons et al., 2018). Furthermore, this might be linked to the type of sexting behavior being analyzed.

Gaining popularity and peer acceptance is one of the main aims of adolescents in their social life (Santor et al., 2000), in face-to-face and online contexts. In fact, research has shown there are no differences between both contexts according to adolescents’ need for popularity (Wright, 2018). It has also been mentioned that there are no gender differences concerning need for popularity (Dijkstra et al., 2010), even when boys and girls could use different strategies to find that popularity: boys increasing the number of sexual partners (Prinstein et al., 2011) and girls taking care of their sexual reputation among peers (Salter, 2016). Need for popularity correlates with sexting participation (Gewirtz-Meydan et al., 2018). Adolescents who feel a stronger need to be popular are more likely to post photos of themselves (Vanden Abeele et al., 2014), thinking that posting their own sexual photos represents a strategic means for them to gain in acceptance among their peers (Baumgartner et al., 2015). From this perspective, the results obtained by Vanden Abeele et al. (2014) indicate that the need for popularity predicts sexting involvement in both, boys and girls.

Need for popularity could also be linked to impact of sexting, as suggested by Alonso and Romero (2019), although maybe not in the same way for boys and girls. Thus, girls, when participating in sexting, receive insults and rejection, experiencing negative feelings post-sexting, negative impact which is not usual in boys (Temple and Choi, 2014; Burén and Lunde, 2018). Need for popularity and gender were also identified as moderators of depressive symptoms (Nesi and Prinstein, 2015), pointing out the potential role of these variables over emotional impact of participants. This suggests that although the need for popularity affects boys and girls, different theoretical models could be required to explain these behaviors (Vanden Abeele et al., 2014).

Taking into account the reviewed literature, our main objective was to analyze the potential emotional impact of sexting as well as the importance of the need for popularity in this phenomenon. Specifically, we sought to examine (1) whether the different types of sexting (primary and secondary) affect those involved in it emotionally; (2) whether the need for popularity is related to both types of sexting and its emotional impact; and (3) whether the aforementioned relationships vary by gender.

In view of the reviewed empirical evidence, our working hypotheses were as follows:

H1: Sexting would have an emotional impact on those involved, but this impact would vary according to the type of sexting and gender.

H2: The need for popularity would affect sexting and its emotional impact but this relationship would vary by gender.

## Materials and Methods

### Participants

The sample comprised 2,356 high school students (46.8% girls, 53.2% boys) from 11 to 18 years of age (*M* = 13.72, SD = 1.31). The participants belonged to 12 compulsory secondary education (ESO) schools, three of which were publicly funded private institutions (*concertados*) from the south of Spain. Specifically, 34.5% were first-year students; 28.7% were second-year students; 21.5% were third-year students; and 14.9% were fourth-year students. However, in order to develop the study of primary and secondary sexting, we used two subsamples. Concretely, the sample for primary sexting was composed by those students who had, or having had, a dating partner in the last 3 months and had sent and/or received sexts at least once. So, this sample was composed of 263 participants (44.5% girls, 55.5% boys; *M* = 14.34, SD = 1.24 years old). The criterion to be part of the subsample of secondary sexting was having forwarded and/or to have been forwarded sexts at least once. Thus, this sample was formed by 621 participants (41% girls, 59% boys; *M* = 14.16, SD = 1.26 years old).

### Measures

Some socio-demographical questions, gender and age, were required. In addition, a direct question about partner was also asked: “Do you have or have you had a partner in the last three months?” with dichotomized answer, (*Yes* or *No*).

To assess sexting, we used four direct questions relating to both primary and secondary sexting involvement, following the guidelines set out in numerous research studies in which direct questions were used to measure involvement (Temple and Choi, 2014; Choi et al., 2016; Gewirtz-Meydan et al., 2018). These behaviors with regard to primary sexting were: *I’ve sent videos, photos or messages of an erotic-sexual nature to my boyfriend/girlfriend* and *I’ve received videos, photos or messages of an erotic-sexual nature from my boyfriend/girlfriend*. With regard to secondary sexting, they were: *I’ve forwarded or shared videos, photos or messages of an erotic/sexual nature of other boys/girls* and *I’ve been forwarded videos, photos or messages of an erotic-sexual nature of other boys/girls*. The responses were formulated using a 5-point Likert scale response format: 0 = Never; 1 = Hardly ever; 2 = Sometimes; 3 = Often; and 4 = Always.

To examine the emotional impact of sexting, an adaptation of the Cybervictimization Emotional Impact Scale, namely the CVEIS (Elipe et al., 2017) was used. Just following the four sexting items, a filter question was asked, and those who said to have sent, received, or forwarded videos, photos, or messages of an erotic-sexual nature were required to fill in the current questionnaire. This questionnaire comprises 18 items that evaluate three types of emotional impact: (1) Active, which includes animated; energetic, lively; satisfied, proud; ready, clear-headed; determined, daring; active, alert; (2) Depressed, which is made up of tense, nervous; guilty; scared, afraid; lonely; ashamed; defenseless, helpless; depressed, sad; fed up; jittery, worried; and (3) Annoyed, which covers angry, annoyed; irritable, in a bad mood; choleric, enraged. If the respondent has engaged in the referred-to phenomenon, he/she should respond by indicating to what extent he/she had experienced each emotion on a Likert scale ranging from 0 = Not at all, to 4 = A lot. Reliability (Rho coefficient) in the present sample was optimal, 0.97 for primary sexting and 0.97 for secondary one, and the results of confirmatory factor analyses (CFA) were adequate: *χ*^2S − B^ = 203.21, *p* = 0.00; CFI = 0.991; NNFI = 0.992; RMSEA = 0.048; SRMR = 0.077 for primary sexting; *χ*^2S − B^ = 334.15; *p* = 0.00, CFI = 0.988; NNFI = 0.986; RMSEA = 0.052, SRMR = 0.090 for secondary sexting.

To assess the need for popularity, we used the *Need for Popularity Scale* (Santor et al., 2000; Utz et al., 2012). This instrument comprises 12 items on a 5-point Likert-type scale (0 = Completely disagree to 4 = Completely agree). Its aim is to evaluate whether behaviors perceived as popular among peers are performed. To this end, items such as *On occasions, I’ve changed the way I dress in order to be more popular* were included. Reliability (Rho coefficient) in the present sample was 0.93, and the confirmatory factor analysis was adequate: *χ*^2S − B^ = 250.33, *p* = 0.00, CFI = 0.991, NNFI = 0.989, RMSEA = 0.043, SRMR = 0.044.

### Procedure

First, we obtained permission from the Andalusia Biomedical Research Ethics Coordinating Committee (0568-N-14), which follows the guidelines set by the International Conference on Harmonization (ICH) Good Clinical Practice (GCP). We then contacted the schools to explain the research to them and request their collaboration. The parental written informed consent has been obtained through the acceptance of participation in the Project that is given by the School Board of each school. In the case of administration of anonymous self-reports related to relevant matters to education, each family, when applying to the schools, delegates, unless expressed otherwise in written, the acceptance of participation to the School Board. This School Board is composed by teachers, students, and representatives of families who behave on behalf of school families. Once the School Board approval had been received, we proceeded to collect data. The questionnaires were administered by specially trained researchers during class time, once teachers had given their prior consent. Completion of the questionnaires took approximately 40 min. Before starting, everyone was informed about the voluntary nature of study participation, response anonymity, and data confidentiality. We stressed the importance of answering truthfully to the students.

### Data Analysis

We ran the analyses for each type of sexting, primary and secondary, with those participants who said to have been involved, at least, hardly ever in these behaviors (sending and/or receiving in primary sexting and forwarding and/or to have been forwarded in secondary one). In addition, primary sexting was assessed just in those who said to have or have had a partner in the last 3 months given that the items in this case were referred to their boyfriend or girlfriend.

First, we performed descriptive analyses (*M*, SD, skewness and kurtosis) of the study variables to explore their distribution as well as to identify potential irregularities, extreme cases, etc., that may skew the results. This was followed by Student’s *t*-tests to analyze possible study variables’ differences between boys and girls. We then tested two structural equation models, one for each type of sexting, and the emotional impact dimensions. After that, we tested the gender invariance of these models. Afterward, we tested two equation models, again one for each sexting type, between need for popularity (NfP from now) and sexting. Once again, gender invariance testing was repeated on these models. Eventually, two more complex models, including NfP, sexting (primary or secondary), and emotional impact were run and, one more time, gender invariance was tested.

The models were estimated *via* the Robust Maximum Likelihood Method, adjusted to the ordinal nature of the study variables (Flora and Curran, 2004). The fit of the models was tested using the following indexes: the Satorra-Bentler scaled chi-square (*χ*^2S − B^) (Satorra and Bentler, 2001); the robust comparative fit index (RCFI) and the non-normality fit index (NNFI) (≥0.90 is adequate; ≥0.95 is optimal); the root mean square error of approximation (RMSEA) and the standardized root mean square residual (SRMR) (≤0.08 is adequate; ≤0.05 is optimal) (Hu and Bentler, 1999). To test the invariance of the models, between-gender multi-group analyses were run. We used a hierarchical strategy. First, we tested a model with no constraints (configural model); second, we tested a model in which equal factor loadings from items to factors were imposed (measurement model); and third, we tested a model in which, besides equal factor loadings, factor variances and covariances were imposed. In order to assess non-invariance, we used the scaled difference chi-square test by Satorra and Bentler (2001). When non-invariance was detected, the Robust Lagrange Multiplier Test (RLMT) was used to analyze which constraints needed to be released in order to achieve invariance. After that, these constraints were released and the new models were run and compared.

The statistical analyses were performed with EQS 6.2. (Bentler, 2006).

## Results

First, we calculated the descriptive statistics of the different study variables and we calculated the Student’s *t*-tests to determine the potential differences between boys and girls (see Table 1). In primary sexting, significant differences were found in active impact, yielding a higher average in boys. In secondary sexting, significant differences in all types of impact were found. Specifically, whereas boys showed a higher average in active impact, the contrary was true for girls in annoyed and depressed impact. No differences were found in NfP in primary and neither secondary sexting according gender (see Table 1).

**Table 1:** Descriptive statistics.

	*M*	SD	Skewness	Kurtosis	Gender	*M* (SD)	*T*	*p*
**Primary sexting (*N* = 263; 44.5% girls, 55.5% boys)**								
Active impact	0.83	1.07	1.17	0.30	Girls	0.52 (0.77)	−4.45	0.00^*^
					Boys	1.09 (1.21)		
Annoyed impact	0.44	0.75	2.38	6.01	Girls	0.46 (0.79)	0.53	0.59
					Boys	0.41 (0.72)		
Depressed impact	0.33	0.78	2.84	8.12	Girls	0.30 (0.74)	0.53	0.59
					Boys	0.36 (0.80)		
Need for popularity	0.75	0.65	1.40	2.24	Girls	0.70 (0.73)	−0.92	0.35
					Boys	0.79 (0.73)		
**Secondary sexting (*N* = 621; 41% girls, 59% boys)**								
Active impact	0.54	0.89	1.82	2.54	Girls	0.25 (0.55)	−7.54	0.00^*^
					Boys	0.74 (1.01)		
Annoyed impact	0.33	0.58	2.66	8.88	Girls	0.39 (0.63)	2.19	0.02^*^
					Boys	0.28 (0.53)		
Depressed impact	0.34	0.76	2.66	7.03	Girls	0.48 (0.91)	3.31	0.00^*^
					Boys	0.25 (0.62)		
Need for popularity	0.70	0.69	1.36	1.97	Girls	0.67 (0.67)	−1.10	0.27
					Boys	0.70 (0.70)		

* *p < 0.05*.

Next, we analyzed two structural equation models with emotional impact. Neither of the models showed adequate statistical adjustment (see Table 2).

**Table 2:** Fit indexes of the models to emotional impact including the three impact factors: Active, Depressed, and Annoyed.

	*χ*^2S − B^	*p*	NNFI	CFI	RMSEA	SRMR
Primary sexting	324.48	0.00	0.85	0.87	0.06	0.09
Secondary sexting	452.25	0.00	0.82	0.84	0.05	0.09

We analyzed then the statistical indexes finding that the contribution of Depressed and Annoyed emotional impact to the models was minimum. In the case of the Annoyed impact, the *R*^2^ was 0.005 for primary sexting and 0.009 for secondary sexting. In the case of the Depressed impact, the *R*^2^ was 0.013 for primary and 0.003 for secondary sexting. Therefore, we decided to test the models again, linking each type of sexting exclusively to the Active impact factor, which has demonstrated a clear relationship to both types of sexting.

**Figure 1:**
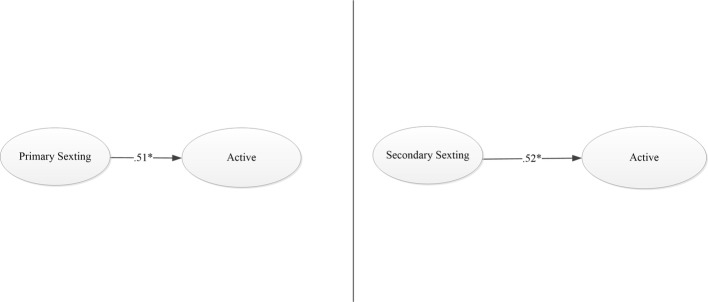
Graphic solution of the sexting models to active impact.

As can be seen in Table 3 and Figure 1, in this case, all models achieved an optimal fit.

**Table 3:** Fit indexes of the models to the active impact.

	χ^2S-B^	*p*	NNFI	CFI	RMSEA	SRMR	*R*^2^
Primary sexting	26.75	0.11	0.98	0.98	0.04	0.02	0.27
Secondary sexting	40.87	0.02	0.96	0.97	0.04	0.03	0.28

Next, we performed an invariance analysis to verify to what extent each of these two models was valid for boys and girls.

Both models showed, in general, gender invariance with the only exception of the most restrictive model, the structural model, in secondary sexting (see Table 4). The RLMT identified equality in sexting as the to-be-released constraint. Once this constraint was released, model showed to be invariant (see Table 4).

**Table 4:** Fit statistics for models of active impact to test gender invariance.

	*Χ*^2^ (Δ*Χ*^2^)	df (Δdf)	*p*	NNFI	RCFI	RMSEA
**Model primary sexting**						
Model 1	102.712	38		0.961	0.973	0.057
Model 2	115.55 (12.84)	44(6)	0.57	0.971	0.978	0.048
Model 3	122.88 (20.16)	46 (8)	0.38	0.967	0.973	0.052
**Model secondary sexting**						
Model 1	185.32	38		0.920	0.946	0.056
Model 2	226.55 (41.24)	44(6)	0.16	0.928	0.943	0.053
Model 3	256.08 (70.77)	46 (8)	0.02	0.912	0.928	0.059
Model 3b	228.57 (43.26)	45 (7)	0.20	0.931	0.945	0.052

*Model 1: Configural invariance. Model 2: Measurement invariance (equal factor loadings). Model 3: Structural model invariance (equal factor loadings, factor variances, and covariances). Χ^2^, Chi square statistic; df, degrees of freedom; NNFI, non-normed fit index; RCFI, robust comparative fit index; RMSEA, root mean-square error approximation*.

**Figure 2:**
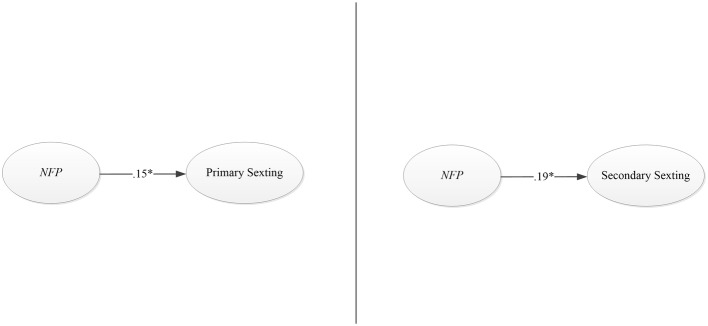
Graphic solution of the models of Need for Popularity (NfP) to sexting.

Two models from NfP to involvement in sexting were then run (See Figure 2). Both showed an optimal adjustment although *R*^2^ was pretty low (see Table 5).

**Table 5:** Fit indexes of the models Need for Popularity to sexting.

	*χ*^2S − B^	*p*	NNFI	CFI	RMSEA	SRMR	*R*^2^
Primary sexting	107.81	0.00	0.92	0.93	0.04	0.05	0.02
Secondary sexting	151.35	0.00	0.92	0.93	0.04	0.04	0.04

The between-gender multi-group analyses showed that these models were totally invariant for girls and boys (see Table 6).

**Table 6:** Fit statistics for the models NfP to sexting to test gender invariance.

	*Χ*^2^ (Δ*Χ*^2^)	df (Δdf)	*p*	NNFI	RCFI	RMSEA
**Model primary sexting**						
Model 1	295.03	152		0.904	0.920	0.046
Model 2	313.61 (18.58)	164 (12)	0.45	0.912	0.920	0.044
Model 3	318.00 (22.97)	166 (14)	0.47	0.914	0.921	0.044
**Model secondary sexting**						
Model 1	396.823	152		0.912	0.926	0.042
Model 2	413.16 (16.33)	164 (12)	0.60	0.920	0.927	0.040
Model 3	414.38 (17.55)	166 (14)	0.72	0.922	0.929	0.040

*Model 1: Configural invariance. Model 2: Measurement invariance (equal factor loadings). Model 3: Structural model invariance (equal factor loadings, factor variances, and covariances). Χ^2^, Chi square statistic; df, degrees of freedom; NNFI, non-normed fit index; RCFI, robust comparative fit index; RMSEA, root mean-square error approximation*.

**Figure 3:**
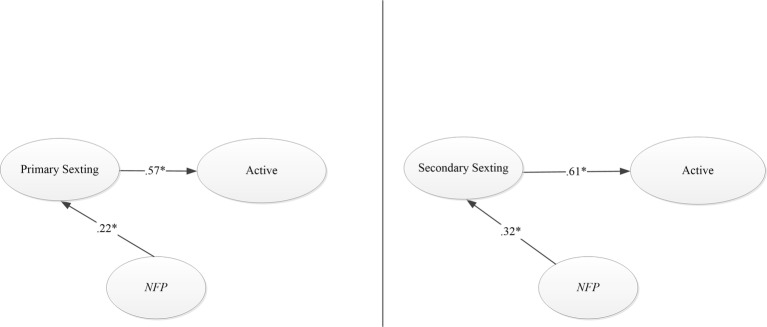
Graphic solution of the models of sexting to active impact including NfP.

Lastly, the models were run by incorporating NfP as a predictor variable of involvement in the different types of sexting (see Table 7 and Figure 3).

**Table 7:** Fit indexes of the models’ active impact and sexting, incorporating the Need for Popularity.

	*χ*^2S − B^	*p*	NNFI	CFI	RMSEA	SRMR	*R*^2^
Primary sexting	235.44	0.00	0.94	0.95	0.04	0.06	0.32
Secondary sexting	272.90	0.00	0.95	0.95	0.03	0.04	0.37

Then, as in the previous cases, next invariance analyses by gender were run revealing, one more time, a total invariance between girls and boys (see Table 8).

**Table 8:** Fit statistics for the models sexting to active impact with NfP to test gender invariance.

	*Χ*^2^ (Δ*Χ*^2^)	df (Δdf)	*p*	NNFI	RCFI	RMSEA
**Model primary sexting**						
Model 1	618,481	336		0.897	0.909	0.053
Model 2	647.48 (28.10)	353 (17)	0.55	0.905	0.912	0.051
Model 3	655.65 (37,17)	356 (20)	0.49	0.906	0.911	0.051
**Model secondary sexting**						
Model 1	768.34	336		0.922	0.931	0.039
Model 2	805.59 (37.25)	353 (17)	0.66	0.930	0.935	0.037
Model 3	810,91 (42,575)	356 (20)	0.70	0.932	0.936	0.037

*Model 1: Configural invariance. Model 2: Measurement invariance (equal factor loadings). Model 3: Structural model invariance (equal factor loadings, factor variances, and covariances). Χ^2^, Chi square statistic; df, degrees of freedom; NNFI, non-normed fit index; RCFI, robust comparative fit index; RMSEA, root mean-square error approximation*.

## Discussion

The overall aim of our study was to advance knowledge of the emotional impact behind sexting, shedding light not only on the emotional impact of this phenomenon among young involved individuals, but also on the influence that the need for popularity has on sexting involvement.

Regarding the first hypothesis, sexting involvement certainly has an emotional impact on those involved. However, this impact does not differ by type of sexting or gender. Irrespective of primary or secondary sexting, it has been determined that this phenomenon does not generate an obvious negative emotional impact at the moment in which the adolescents engage in sexting practices. Although the phenomenon has a clear emotional impact on adolescents, at least in the short term during involvement, this impact is linked to the active emotions (I feel lively, energetic, satisfied, ready, determined, active).

Thus, contrary to studies that observed a correlation between sexting and negative aspects including anxiety and depression (Strasburger et al., 2012; Jasso Medrano et al., 2018), in our study the emotions generally considered “negative,” that is, those related to sadness and depression, and those related to anger or annoyance, were not associated with sexting involvement. These results seem to support the line of argument held by some authors that sexting itself is not the cause of negative emotional impact; rather, it could be further episodes, such as the non-consensual dissemination of images to third parties, a rapidly increasing target audience, and social judgments upon the victim’s reputation, that would likely cause this damage (Livingstone and Görzig, 2014; Van Ouytsel et al., 2014b). It is important to bear in mind that we have assessed the impact of sexting involvement in first person (I’ve sent, I’ve received, I’ve forwarded, I’ve been forwarded), and we have not elicited any information by way of question about whether the personal content sent has been distributed to third parties. It is highly likely that the negative consequences referred to in previous studies are a result of this situation (Livingstone and Görzig, 2014).

From this perspective, the fact that sexting generates an active emotional impact, as opposed to depressive or anger-based responses linked to poor psychosocial adjustment, could be something positive. However, this very aspect may also represent a risk to adolescents (Crockett et al., 2006). The active emotional response may act as a stimulus that invites adolescents to not anticipate and assess – or either manages to minimize – the possible effects of primary and secondary sexting and the associated risks. Therefore, experiencing a positive emotional impact when engaging in sexting can indicate a lack of awareness of the potential consequences of this practice. This highlights the need to explore this issue further by focusing on those actions whose aim is to prevent and address sexting.

In terms of gender differences, the results obtained suggest that the emotional impact behind both types of sexting is similar in boys and girls. On the basis of the findings of this study, it is possible to speculate that while girls could experience higher social pressure to engage in sexting, as reported in some studies (Wood et al., 2015), this pressure, be it perceived that way or not, does not translate into anger, as one might expect, but into activation. From this perspective, it would be interesting to qualitatively analyze what interpretation is made of the cited pressure. It might also indicate that those involved in these behaviors do not perceive a risk of potential forwarding thereafter, and as suggested by Ybarra and Mitchell (2014), they conceive sexting as a romantic and enriching part of the relationship, although it carries an element of danger.

Although depressed and anger dimensions of emotional impact were not relevant in models, and these models showed invariance between genders, we should not forget girls presented significantly higher scores in depressed and anger dimensions of emotional impact when we analyzed secondary sexting, and boys have a significantly higher active emotional impact in both types of sexting. In line with Ringrose et al. (2013), these results point to a different meaning behind sexting involvement by gender, reinforcing the idea of a double sexual standard to explain different consequences for boys and girls (Symons et al., 2018). This different impact can also be linked to other variables like motivations of sexting (Bianchi et al., 2018), non-consensual participation in sexting (Dekker and Thula, 2017), or some other factors associated to sexting experience like social pressure or threat (Lee and Crofts, 2015).

Regarding the second hypothesis, the need for popularity has contributed to understand the implication in sexting, but even more to explain the emotional impact of both types of sexting, slightly more in the case of the secondary one. As we used a scale to assess need for popularity in offline context, this reinforces the continuity between face-to-face and online worlds, as Wright (2018) suggested. When need for popularity is included in structural models of emotional impact of sexting, it increases their goodness fit. This coincides with previous studies which indicate that sexting participation could be linked to the need for popularity (Gewirtz-Meydan et al., 2018). Adolescents who feel a greater need for popularity are far more likely to post photos of themselves (Vanden Abeele et al., 2014), taking the view that posting their own sexual photos is a strategic way of gaining popularity among peers (Baumgartner et al., 2015).

As for gender differences, the explanatory power of the need for popularity in the emotional impact generated by primary and secondary sexting is similar in boys and girls. These results demonstrate that seeking peer acceptance through popularity is an important motivation for boys and girls when it comes to participating in these practices. From this perspective, the results obtained by Vanden Abeele et al. (2014) indicate that the need for popularity predicts, in a similar way, sexting involvement by boys and girls. Both sexes would seek acceptance and popularity by engaging in sexually permissive behaviors. However, the consequences of these practices would vary among them. These practices would help boys enhance their social capital and be more accepted within their peer group. In contrast, girls would, for the most part, not only gain in popularity but also be at the receiving end of insults and rejection, having their reputation damaged and experiencing negative feelings (Ringrose et al., 2013; Temple and Choi, 2014; Wood et al., 2015; Burén and Lunde, 2018). This reality echoes the sexual double standard that seems to govern this phenomenon (Wood et al., 2015; Symons et al., 2018).

## Conclusions

Our study presents novel findings on the emotional impact of sexting and the influence of the need for popularity on adolescents. Taken together, the results reveal sexting to be a phenomenon that, in itself, does not appear to generate a negative impact among those involved, in a short term. Its impact, which is essentially “active,” seems to more strongly correlate with typical behaviors of desire and curiosity about new experiences, much like the sexual experiences that play out during this developmental stage. The importance attached to the need for popularity when studying sexting behavior involvement has also been confirmed, given that adolescents who feel the need to be popular may see the exchange and distribution of sexual content as a strategy for gaining in acceptance into the peer group.

No significant gender differences were observed for either the emotional impact of sexting or the explanatory power of the need for popularity in the impact of primary and secondary sexting. However, girls presented significant higher scores when we analyzed depressed and anger dimensions in the case of secondary sexting. This finding invites us to continue exploring the role of sexting not only in terms of interaction with potential dating partners, but also in terms of female and male group status. This knowledge is essential as it enables us to identify key areas for designing prevention and intervention proposals that address sexting.

Our study does, however, pose some limitations that warrant mention. Measures applied in this study have shown some restrictions linked to a developing topic. In this sense, to our knowledge, no previous instruments have been applied to assess emotional impact in sexting episodes, and need for popularity has been usually assessed asking only for offline context. Additionally, we did not assess whether the content sent had been distributed to third parties without the sender’s prior consent, or about the different motivations which could be behind sexting. Including these variables could be an important aspect for exploring the consequences of sexting further. That said, this is the first study to analyze the emotional impact of sexting, and we need to continue along these lines of inquiry.

As a future line of research, it would be useful to conduct qualitative studies that allow us to capture and analyze gender differences in greater depth. It is possible that some of the differences to emerge from the medium- and long-term consequences among boys and girls have more to do with the socially attributed meaning given by the protagonist of the sexual material, be it male or female, than with the impact brought about by the undertaking of sexting behavior itself.

## Data Availability

The datasets generated for this study are available on request to the corresponding author.

## Ethics Statement

The study was approved by the Comité Coordinador de Ética de la Investigación Biomédica de Andalucía (Coordinating Ethics Committee of Biomedical Research of Andalusia) and was in accordance with all regulations concerning professional ethics as stated in the International Conference on Harmonization Good Clinical Practice Guideline. The study was approved by the school boards and the students were visited and the anonymous, confidential, and voluntary nature and the objective of the study were explained before the survey was taken.

## Author Contributions

RR and JM-M designed the study. RR, JC, and MO collected the data. JC and PE designed and conducted the statistical analyses in close consultation with RR, MO, and JM-M. MO and RR wrote the first draft of the introduction, PE and JC wrote the first draft of the method and results, and JM-M wrote the first draft of the discussion, in close consultation of all the authors. All authors contributed to and have approved the final manuscript.

### Conflict of Interest Statement

The authors declare that the research was conducted in the absence of any commercial or financial relationships that could be construed as a potential conflict of interest.
